# Implications of Tributyrin on Gut Microbiota Shifts Related to Performances of Weaning Piglets

**DOI:** 10.3390/microorganisms9030584

**Published:** 2021-03-12

**Authors:** Francesco Miragoli, Vania Patrone, Aldo Prandini, Samantha Sigolo, Matteo Dell’Anno, Luciana Rossi, Alice Senizza, Lorenzo Morelli, Maria Luisa Callegari

**Affiliations:** 1 Department for Sustainable Food Process (DiSTAS), Università Cattolica del Sacro Cuore, via E. Parmense 84, 29122 Piacenza, Italy; francesco.miragoli@aat-taa.eu (F.M.); vania.patrone@unicatt.it (V.P.); alice.senizza@unicatt.it (A.S.); lorenzo.morelli@unicatt.it (L.M.); 2 AAT—Advanced Analytical Technologies Srl, Fiorenzuola d’Arda, 29107 Piacenza, Italy; 3 Department of Animal Science, Food and Nutrition (DIANA), Università Cattolica del Sacro Cuore, via E. Parmense 84, 29122 Piacenza, Italy; aldo.prandini@unicatt.it (A.P.); Samantha.sigolo@unicatt.it (S.S.); 4 Department of Health, Animal Science and Food Safety, Università degli Studi di Milano, via Trentacoste 2, 20134 Milano, Italy; matteo.dellanno@unimi.it (M.D.); luciana.rossi@unimi.it (L.R.)

**Keywords:** weaning, piglets, gut microbiota, tributyrin, animal performance

## Abstract

Alternatives to antibiotic treatments are required owing to the ban on the use of these drugs as growth promoters in food animal production. Tributyrin appears to play a role in improving growth performance in pigs, albeit with varying degrees of effectiveness. So far, very little is known about its effects on gut microbiota composition. In this study, we investigated the gut microbiota changes of piglets receiving, at weaning, 0.2% tributyrin added to their basal diet. Microbiota composition was assessed through 16S-rRNA gene sequencing on stools collected from tributyrin and control groups. The functional profiles of microbial communities were predicted from amplicon abundance data. A comparison between dietary groups revealed that tributyrin strongly modulated gut microbiota composition in piglets, increasing the relative abundance of a number of bacterial genera such as *Oscillospira*, *Oscillibacter*, *Mucispirillum* and *Butyrivibrio*. These genera were positively correlated to animal average daily gain (ADG) and/or body weight (BW). Based on the function profile prediction, the gut microbiome of the tributyrin group possessed an enhanced potential for energy metabolism and a reduced potential for carbohydrate metabolism. In conclusion, our results indicated that tributyrin can promote changes to gut microbial communities, which could contribute to improving animal performance after weaning.

## 1. Introduction

Early weaning represents a practice that offers economic benefits for farmers, and for this reason, it is adopted by most intensive breeding practitioners in developed countries. Weaning is a period in the life of piglets characterized by several stressing factors that lead to reduced performance and contribute to the development of intestinal dysfunctions such as postweaning diarrhea. It is well known that, during weaning, several factors shape the gut microbiota composition [1]. Among these, changes of diet represent one of the most impactful conditions [2] and can be related to diarrhea. Other factors, such as infectious agents [3,4,5] and the weaning-mediated evolution of the gut microbiota [6,7,8] are also responsible for the development of these intestinal diseases.

Antibiotic treatments still represent the only strategy for controlling diseases related to weaning, though a series of feed supplements have been proposed as effective alternatives to drug treatments [9,10,11] following the ban of food animal growth-promoting antibiotics by the European Union in 2003 (EC No 1831/2003) [12]. In this context, tributyrate glyceride, also called tributyrin, appears to be a good candidate for alleviating intestinal dysfunctions, modulating immune response and improving growth performance in piglets. Despite the large number of papers addressing tributyrin impact on weaning piglets, its efficacy as a growth promoter is sometimes controversial [13,14,15,16] probably because of different experimental conditions in farm trials. Indeed, the efficiency of tributyrin, as with all other growth promoter candidates, may vary based on its mechanisms of action, the basal diet composition and the animal’s health conditions. Despite several studies concerning butyric acid and its derivatives, their mechanisms of action are still unclear. Morphological changes of the intestinal mucosa are one of the impacting factors on the animal’s welfare, and some studies indicated that tributyrin could alleviate these weaning-related symptoms. In particular, tributyrin demonstrated effectiveness in modifying crypt depth and villus height in different intestinal sections [13,15,16,17,18].

Thus, all these results suggest that tributyrin supplementation, albeit with varying degrees of effectiveness, plays a role in alleviating the detrimental effects on gastrointestinal barrier properties caused by early weaning that can lead to inflammatory status. Moreover, Gu et al. [19] have reported an anti-inflammatory action of tributyrin in piglets as a consequence of the intraperitoneal injection of *Escherichia coli* lipopolysaccharide (LPS), a model of enteropathogenic infections. Another proposed mechanism of tributyrin action is the reduction in reactive oxygen species (ROS) [20] and the protection of mitochondrial function against oxidative stress [17].

Antimicrobial activities of organic acids and their derivatives, including monobutyrin, have been reported in an in vitro study [21]. The antimicrobial effects of butyrate glycerides on *Salmonella Typhimurium* and *Clostridium perfringens* were studied in poultry [22]. However, there is little information regarding the effects of tributyrin on pigs’ intestinal microbiota, with most of the studies being concerned with bacteria considered as an indicator of animal health [14,16]. In our previous study [14], we identified significant differences in body weight (BW), average daily gain (ADG) and several blood parameters in piglets receiving tributyrin during the weaning period, as compared to the control. In that study, we specifically addressed the impact of tributyrin on lactobacilli and bifidobacterial, although other gut bacterial populations have been associated with high feed efficiency in pigs [23,24]. The aim of the present work was thereby to comprehensively assess the gut microbiota composition of pigs fed tributyrin by means of high-throughput 16S-rRNA gene sequencing. In the present work, the fecal samples from the aforementioned animal trial were exploited to gain insight into the structure and metabolic potential of gut microbial communities, along with the production of short-chain fatty acids (SCFAs). Microbiological data were correlated with physiological and performance parameters measured in the previously mentioned work, in order to investigate how changes to gut microbiota could impact both animal health and performance.

## 2. Materials and Methods

### 2.1. Animals and Sample Collection

The animals used in the present study were selected among those involved in the trial described by Sotira et al. [14]. For the full details concerning animal experimentation and ethics, basal diet composition, breeding type and experimental design, we refer the reader to this previous publication. The trial was approved by the animal welfare body of University of Milan (authorization number 31/2019) according to Italian regulation on animal experimentation and ethics (DL 26/2014) and to European regulation (Dir. 2010/63). All procedures were carried out according to relevant guidelines and regulations. Briefly, 120 weaned piglets (28 ± 2 days) were allotted in a randomized complete block design into two experimental groups: control (CTR) and tributyrin (TRI). Each group constituted 60 pigs (6 replicate pens with 10 pigs per pen). The groups were homogeneous for gender, weight and litter. Furthermore, the two groups were raised in the same room with a free floor surface where temperature (27 °C) and humidity (60%) were controlled. Water and feed were supplied ad libitum. The area available for each animal was 0.40 m^2^, according to the Directive 2008/120/EC. After one-week of adaptation in which the animals received the basal diet (corresponding to the day 0 and 35 days from birth), experimental diets were fed ad libitum for each group for 40 days. The CTR group received the basal diet whereas the TRI group received the same basal diet supplemented with 0.2% of tributyrin (ACIFIS^®^ Tri-B, New Feed Team srl, Lodi, Italy). The experimental diets were isoenergetic and isoproteic and complied with the NRC requirements [25] for postweaned piglets, as already reported by Sotira et al. [14] (Table S1). Two animals per pen were then randomly selected for each treatment group for faecal sample collection. At day 40, corresponding to 75 days from the birth of piglets, stool samples were individually collected from rectal ampulla and immediately frozen in dry ice. Once transferred to the laboratories, they were stored frozen at −80 °C until DNA extraction.

### 2.2. DNA Extraction, 16S rRNA Gene Amplification and Sequencing

Total bacterial DNA was extracted from 50 mg (wet weight) using the FastDNA™ SPIN Kit for Soil (MP Biomedicals, Eschwege, Germany), following the manufacturer’s instructions. The genomic DNA was then quantified using the Qubit HS dsDNA fluorescence assay (Life Technologies, Carlsbad, CA, USA), while the quality of the extracted DNA was assessed using agarose gel electrophoresis. DNA amplification was performed using primers 343F and 802R, which allowed the amplification of the V3–V4 regions of the bacterial 16S rRNA gene. Amplification was carried out as described above [26]. The PCR products were then analyzed using agarose gel electrophoresis and quantified using the Qubit HS dsDNA fluorescence assay (Life Technologies, Carlsbad, CA, USA). Finally, a pool of amplicons was prepared by adding the PCR products of each sample in an equimolar concentration and then purified using a DNA Clean & Concentrator™-5 Kit (Zymo Research, Irvine, CA, USA).

Sequencing was performed at Fasteris SA (Geneva, Switzerland) using Illumina’s MiSeq v3 platform with 2 × 300 bp mode. Raw reads were filtered for low quality (quality score ≥ 30) by Trimmomatic tool version 0.32 (http://www.usadellab.org/cms/index.php?page=trimmomatic, accessed on 26 August 2020) [27] by sliding window trimming (window size: 4 bases, quality: 15). Reads showing a length shorter than 60 bases were excluded. Overlapped reads were mapped against the SILVA database (Version SSURef_NR99_115_tax_silva_DNA.fasta) using Burrows–Wheeler Alignment Tool version 0.7.5a (http://bio-bwa.sourceforge.net/, accessed on 28 August 2020) [28]. The SSU dataset was composed of high-quality 16S/18S rRNA sequences (99% criterion) applied to remove redundant sequences. In order to merge alignments and to compute the number of reads onto each OTU, the SAM tools package was used [28]. Sequence files were deposited in the European Nucleotide Archive (ENA) database under the accession number PRJEB40653.

### 2.3. Gas-Chromatographic Analysis of Short-Chain Fatty Acids

Short-chain fatty acids (SCFAs) were extracted from a 3 g sample dissolved in 9 mL of distillate water by stirring for a few minutes. Following centrifugation at 480× *g* for 15 min, 2 mL of supernatant was added to 1 mL of a pivalic acid solution (internal standard, 1 g L^−1^ in distilled water) and 1 mL of a 0.12 M oxalic acid solution. This suspension was mixed using a vortex and then centrifuged at 480× *g*. The upper phase was microfiltered.

The gas-chromatographic analysis was carried out using a Shimadzu 2025 gas chromatograph equipped with an AOC-20i autosampler (Shimadzu Srl, Milan, Italy), an FID detector and a 30 m × 0.250 mm, 0.25 µm DB-FFAP capillary column (Agilent Technologies, Inc. Santa Clara, CA, USA). The injector and detector temperatures were 200 and 220 °C, respectively. The injection was carried out in split mode (1:100), and a volume of 1 µL was injected. The analysis was carried out at a constant flow of hydrogen gas using a programmed temperature: the initial temperature was 60 °C and held for 5 min. The temperature was then raised to 160 °C at 5 °C min^−1^ and finally to 190 °C at 10 ° C min^−1^ and held for 7 min.

Data acquisition and processing were conducted using the LabSolutions Lite software (version 5.82, Shimadzu Srl, Milan, Italy). SCFA identification was based on the retention time of the volatile free acid mix standard (Sigma-Aldrich, St. Louis, MO, USA), used as the external standard. Pivalic acid was used as the internal standard.

### 2.4. Statistical Analysis

The statistical analysis of the sequences was performed using the MicrobiomeAnalyst tool [29,30]. Alpha diversity was calculated based on Chao, Observed species, Simpson and Shannon metrics, and significant differences were calculated using a *t* test ANOVA. Beta diversity was determined via the Bray–Curtis index using a PERMANOVA statistic method, and community beta diversity across samples was visualized using a Principal coordinate analysis (PCoA) plot. Significant differences in the taxa abundance between the two groups of samples were identified using the edgeR algorithm with 0.05 adjusted *p*-value cut-off. In order to predict, in silico, the functional profile of the gut microbial populations across the two dietary groups, the Marker Data Profiling tool included in the MicrobiomeAnalyst software was used. The purpose was to generate a corresponding KEGG Onthology (KO) assignment table using the Tax4Fun source. The KO table was then uploaded via the Shotgun data profiling option in order to analyze the differential relative abundance of genes across the two dietary groups. A Cluster of Orthologous Groups (COG) of proteins table, obtained from the same tool, was analyzed using the EdgeR algorithm, with a 0.05 adjusted *p*-value cut-off.

GraphPad Prism v.5 (Graphpad Software, San Diego, CA, USA) was used to perform the t test analysis of short-chain fatty acid concentrations in order to underline the differences between the two groups of animals. Differences were considered significant when *p* < 0.05. Spearman’s correlation analysis was performed in order to test the association between performances and physiological parameters across the two dietary groups of animals using GraphPad Prism v.8 (Graphpad Software, San Diego, CA, USA). The corresponding heatmap and table containing both *r* and *p* values were automatically generated.

## 3. Results

### 3.1. Microbiota Composition and Community Diversity Associated with Tributyrin Supplementation

**Figure 1:**
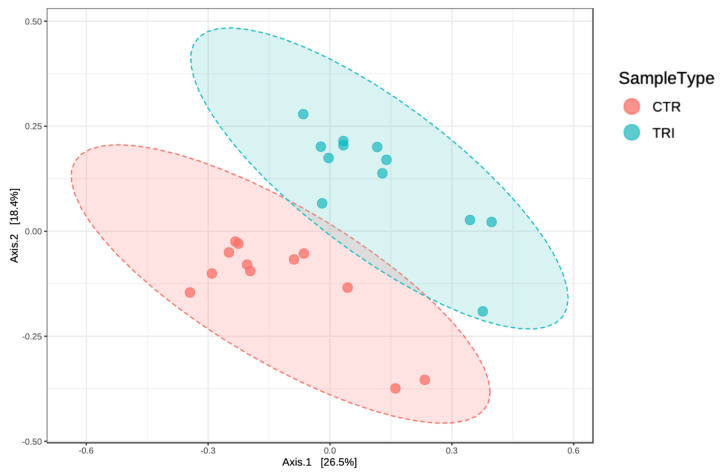
Principal coordinates analysis (PCoA, Bray-Curtis distance) plot of the gut microbiota of weaning piglets fed a diet with (TRI) or without tributyrin supplementation (CTR) (R2 = 0.19, *p* < 0.01).

The total number of filtered sequences obtained in the samples collected from tributyrin and control animal groups was 2,405,009. The median sequencing coverage was 100,209 sequences per sample with minimal and maximal coverages of 65,975 and 171,531 reads, respectively. Based on principal coordinates analysis (PCoA), we observed significant diet-specific differences in gut bacterial communities (Figure 1), with the supplementation of tributyrin accounting for about 20% of the total variance (Permanova analysis, R^2^ = 0.19, *p* < 0.01).

Tributyrin supplementation had no significant influence on fecal bacterial alpha diversity calculated by Chao 1 (*p* = 0.195), observed OTUs (*p* = 0.102), Simpson (*p* = 0.737) and Shannon (*p* = 0.144) indices. These results indicated that no significant differences were observed in term of species diversity and richness among dietary groups.

**Figure 2:**
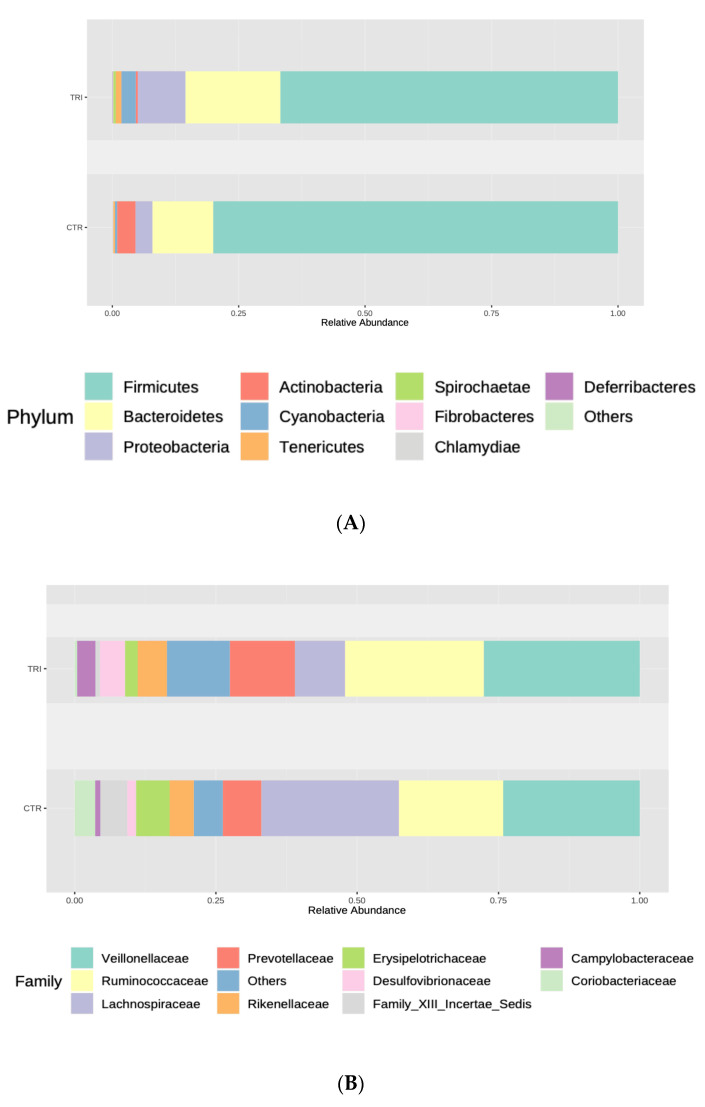

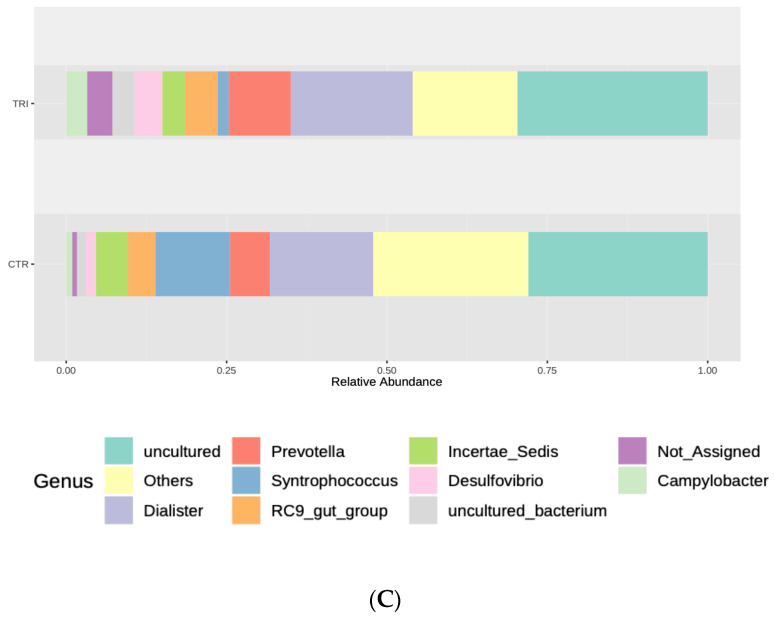
Relative abundances of the different phyla (**A**), families (**B**) and genera (**C**) observed in the tributyrin animal group (TRI) compared with those of the control group (CTR). Only top taxa are shown.

At the phylum level, the fecal microbiota collected from the control group was dominated by *Firmicutes* (77.97%) followed by *Actinobacteria* (13%) and *Bacteroidetes* (6.52%), whereas animals receiving tributyrin supplementation showed a microbiota dominated by *Firmicutes* (73%) followed by *Bacteroidetes* (15%) and *Actinobacteria* (1%) (Figure 2A). In this group, the relative abundance of *Firmicutes* and *Actinobacteria* decreased significantly in fecal samples (FRD = 0.002 and FDR = 0.0235, respectively), while a significant increase was detected in the levels of *Proteobacteria* (FDR = 0.0150). Additionally, the relative abundance of *Cyanobacteria*, *Deferribacteres* and *Spirochaetae* (Supplementary Table S2) increased in tributyrin-fed piglets.

At the family level, *Desulfovibrionaceae*, *Mycoplasmataceae*, *Alcaligenaceae*, *Campylobacteriaceae*, VadinBB60 and *Deferribacteraceae* proved to be significantly enriched in samples collected from the tributyrin group. In addition, the tributyrin supplementation caused significant reductions in the relative abundances of *Coriobacteriaceae*, *Peptococcaceae*, *Peptostreptococcaceae*, *Erysipelotrichaceae*, *Lachnospiraceae* and Family XIII *Incertae Sedis* (Figure 2B and Supplementary Table S2).

Moreover, at the genus level, significant increases in Oscillospira, Desulfovibrio, Pseudoflavonifractor, Butyrivibrio, Sutterella, Oscillibacter, Mycoplasma, Campylobacter, Anaerotruncus, Mucispirillum and the RC9 gut group genera was found in the tributyrin group samples. In addition, Mogibacterium, Collinsella, Peptococcus, Atopobium, Subdoligranulum, an uncultured bacterium, Syntrophococcus, Marvinbryantia, Blautia, Enterorhabdus, Oribacterium and Denitrobacterium genera levels were significant reduced following tributyrin supplementation (Figure 2C and Supplementary Table S3).

**Figure 3:**
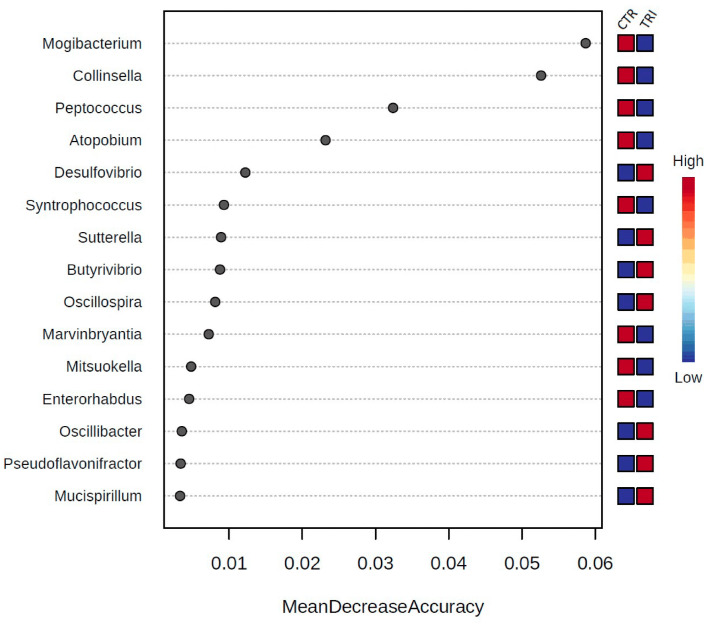
The 15 most discriminant genera between control and tributyrin sample sequences as determined by Random Forest analysis using mean decrease in accuracy.

Moreover, Random Forest analysis revealed that at the family level, *Coriobacteriaceae* and Family XIII *Incertae Sedis* were the most important features in discriminating between the two groups of animals. Among the members of the aforementioned families, the genera *Mogibacterium*, *Collinsella*, *Peptococcus* and *Atopobium* were the most important discriminant genera, since their representative points are on the right and at the top of the graph, indicating their higher predictive values (Figure 3).

### 3.2. Functional Prediction

**Figure 4:**
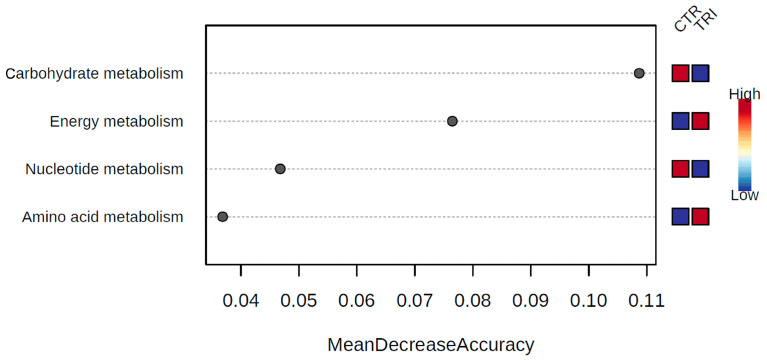
The most discriminant Cluster of Orthologous Groups (COG) functional categories between control and tributyrin samples determined by Random Forest analysis using mean decrease in accuracy.

The operational taxonomic unit (OTU) table obtained through sequence analysis was uploaded to the MicrobiomeAnalyst software using the Marker Data Profiling option. The Kegg Onthology assignment table was generated using Tax4Fun and was then used for further analysis via the Shotgun data profiling option provided by the same software. Random Forest analysis revealed that the most discriminant COG functional categories between the two groups of samples were carbohydrate and nucleotide metabolisms, more prominently represented in the control group, whereas energy and amino acid metabolisms were enriched in the tributyrin group (Figure 4). Based on EdgeR analysis, only the energy metabolism pathways were found to be significantly increased (FDR = 0.007) in the tributyrin group, combined with a significant reduction in carbohydrate metabolism (FDR = 0.007). In particular, in the control group, we found enriched genes encoding sugar transport, mainly components of phosphotransferase systems (K02768, K02769, K02793, K02795 and K02796). Conversely, in the tributyrin samples, genes encoding amino acid utilization as an energy source (K00830, K00830 and K02204) were enriched, compared to the control group. Other genes were identified as being more prominently represented in the samples of the tributyrin group, some of which were related to gluconeogenesis (K03841 and K16153) and others to the glyoxylate cycle (K00122, K01638, K00283 and K00605).

### 3.3. SCFA Concentration in Stools

The SCFA concentration in the stools of both dietary groups of animals was recorded. The most abundant SCFAs were acetate, propionate and butyrate, which did not reveal significant differences between the two animal groups, along with isovalerate and valerate. However, the tributyrin group of piglets revealed a 1.31-fold increase in their fecal concentration of isobutyrate, and this difference between the two groups of animals was statistically significant (*p* = 0.0269) (Table 1).

### 3.4. Correlation between Microbiota and Piglet Physiological/Performance Parameters

**Figure 5:**
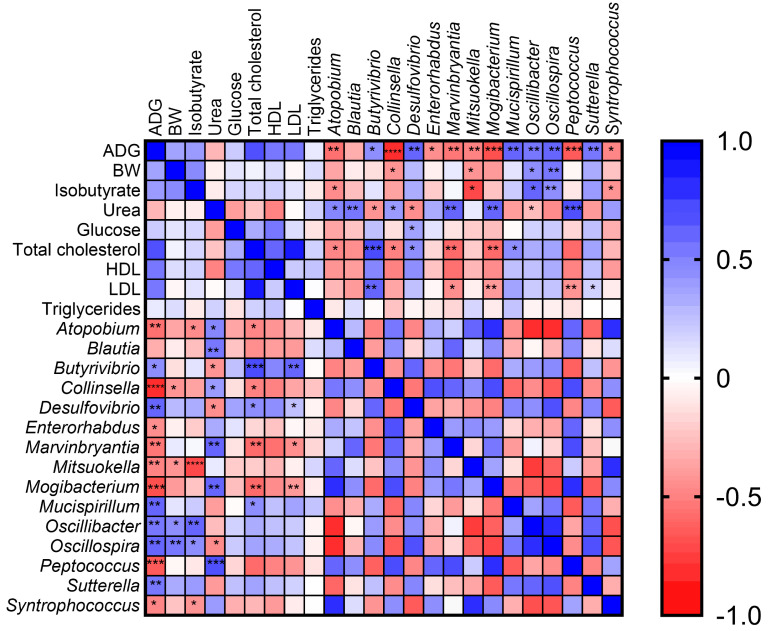
The Spearman correlation heatmap graphically represents the correlation between Average Daily Gain (ADG), Body Weight (BW), isobutyrate, urea, total cholesterol, High-Density Lipoproteins (HDLs), Low-Density Lipoproteins (LDLs) and significant different genera between the two dietary groups. The colors display the *r*-value. * *p* ≤ 0.05; ** *p* ≤ 0.01; *** *p* ≤ 0.001 and **** *p* ≤ 0.0001. *p* value significances were graphically reported only for correlation between the aforementioned parameters and bacterial genus.

Spearman’s correlations were calculated between the physiological and performance data evaluated in our previous work and significant genera were found via statistical analysis. The correlation results are graphically presented in Figure 5, while the detailed data are presented in Supplementary Table S4.

Positive correlations were found between *Butyrivibrio* (*r* = 0.45, *p* = 0.03), *Desulfovibrio* (*r* = 0.59, *p* = 0.002), *Oscillibacter* (*r* = 0.53, *p* = 0.007), *Sutterella* (*r* = 0.54, *p* = 0.007) and ADG. Conversely, *Atopobium* (*r* = −0.56, *p* = 0.005), *Collinsella* (*r* = −0.81, *p* = 0.000002), *Enterorhabdus* (*r* = −0.44, *p* = 0.03), *Marvinbryantia* (*r* = −0.53, *p* = 0.007), *Mitsuokella* (*r* = −0.49, *p* = 0.015), *Mogibacterium* (*r* = −0.68, *p* = 0.0003), *Peptococcus* (*r* = −0.65, *p* = 0.0006) and *Syntrophococcus* (*r* = −0.47, *p* = 0.019) revealed negative correlations within the aforementioned animal parameters. Moreover, *Oscillibacter* (*r* = 0.44, *p* = 0.03) and *Oscillospira* (*r* = 0.55, *p* = 0.0056) were also positively correlated with animal BW.

Interestingly, a positive correlation was found between isobutyrate and both *Oscillibacter* (*r* = 0.57; *p* = 0.003607) and *Oscillospira* (*r* = 0.60, *p* = 0.0017), whereas *Atopobium* (*r* = −0.45; *p* = 0.026)*, Collinsella* (*r* = −0.37; *p* = 0.07), *Mitsuokella* (*r* = −0.71; *p* = 0.00009) and *Syntrophococcus* (*r* = −0.42; *p* = 0.043) were negatively correlated.

*Atopobium* (*r* = 0.48; *p* = 0.017), *Blautia* (*r* = 0.53; *p* = 0.007), *Collinsella* (*r* = 0.42; *p* = 0.043), *Marvinbryantia* (*r* = 0.60; *p* = 0.0020), *Mogibacterium* (*r* = 0.62; *p* = 0.0013), and *Peptococcus* (*r* = 0.67; *p* = 0.0003) were positively correlated with urea concentration values. Conversely, negative correlations were found between urea and *Butyrivibrio* (*r* = −0.43; *p* = 0.037) and *Desulfovibrio* (*r* = −0.44; *p* = 0.032).

## 4. Discussion

The primary aim of this work was to investigate the effects of tributyrin on the gut microbiota of weaned piglets. Secondly, we focused our attention on how the composition of these communities could be associated with animal performances. Using 16S rRNA gene sequencing, we detected a number of compositional changes of fecal bacterial populations indicating that tributyrin substantially altered the gut microbial community of piglets. *Actinobacteria* was the most significantly decreased phylum in tributyrin-fed animals, driven by a significant reduction in the family *Coriobacteriaceae* and the genera *Collinsella* and *Atopobium*. In addition, Random Forest analysis revealed that the most discriminant genera between the two dietary groups of animals were *Mogibacterium*, *Collinsella*, *Peptococcus*, and *Atopobium*. All these genera decreased in the tributyrin group compared with controls. Little information regarding these genera is available. *Collinsella* is known to establish persistent colonization of intestinal mucosa via utilization of mucins in both pigs and humans [31,32,33]; its close association with the mucus layer suggests a direct interaction between these microorganisms and the host’s intestinal tissues. A decrease in the relative abundance of *Collinsella* has been previously described in several studies [34,35,36] as being associated to reduced gut permeability and hence to an improved functionality of the intestinal barrier. *Mogibacterium*, a genus belonging to the *Clostridium Family XIII Incertae Sedis*, has been shown to increase in mucosa-associated microbiota of colon cancer patients [37] but to decrease, together with *Collinsella*, in the feces of neonatal pigs administered with a beneficial prebiotic preparation [38]. Burrough et al. [39] reported a high relative abundance of *Mogibacterium* in both mucosal scrapings and luminal samples from pigs with swine dysentery. *Mogibacterium* spp. produce phenyl acetate as their unique final metabolic product [40] and they have been associated with ammonia assimilation in the rumen of cows [41]. Tributyrin supplementation also resulted in lower numbers of *Peptococcus*; this taxon has been often detected in the gut of pigs and its levels have been reported to be negatively associated with feed conversion ratio [42] but positively correlated with preweaned weight gain [43]. Based on our correlation results, *Collinsella* displayed a strong negative correlation with both ADG and BW, suggesting a potential relationship between this population and pig performance. Our results are in line with those of Kubasova et al. [44], who showed that the microbiota of high-residual feed intake pigs were enriched in *Collinsella* compared with animals that were more efficient. *Mogibacterium*, *Peptococcus* and *Atopobium* correlated negatively to ADG parameter as well. A positive correlation between *Mogibacterium* and blood urea concentration was also found; from such a result, we can only speculate that the decrease in this genus in tributyrin-fed animals is somewhat linked to the decrease in plasma urea previously described.

The higher abundance of *Proteobacteria* observed in the stool of tributyrin-fed piglets was driven by a significant increase in the *Desulfovibrio* genus. Indeed, this could be related to an increased hydrogen production in the lumen, as the growth of members of this genus is highly dependent on this gas. Since *Desulfovibrio* species are hydrogen consumers, they play a key role in removing this fermentation inhibitor from the intestinal lumen [45]. This important role in the progression of fermentation could explain the positive correlation of *Desulfovibrio* with the ADG parameter we identified.

In addition to the above-described populations, other bacterial taxa resulted differentially present in the gut microbiota of tributyrin-fed piglets [46]. Most of the genera that are significantly increased in the tributyrin group have been described in previous works as enriched in high-feed efficiency pigs. In particular, McCormack et al. [23] reported that, in weaning piglets, the *Butyrivibrio* genus was present only in animals showing high performances, which could be due to the enhanced ability of their gut microbiota to ferment complex carbohydrates. In the same study, the *Oscillibacter* genus was associated with improved weight gain [47]. Both *Butyrivibrio* and *Oscillibacter* were the genera that increased mostly significant in the tributyrin group, along with *Mucispirillum*. Likewise, the latter genus showed higher relative abundance in low residual feed intake (RFI) animals [24] and was found to be present uniquely during the postweaning phase. *Mucispirillum* is an opportunistic mucin utilizer that has been identified as part of an immunogenic commensal group in humans. This genus adheres to the intestinal mucus and interacts with the host immune system; through a T-dependent IgA response, they never become dominant [48]. This information is reassuring with regard to the role of *Mucispirillum,* since it has sometimes been assumed to be harmful to the integrity of the mucus layer [49]. Our results seem to confirm these positive roles played by *Butyrivibrio* and *Mucispirillum* in animal performances, as indicated by their positive correlations with ADG values. Moreover, *Oscillibacter* positively correlated with BW. As for *Oscillospira,* species belonging to this genus are capable of using alternative energy sources such as mucins and glucoronate [50]; the reduced number of sugar transporter genes in their genomes has been correlated to their inability to use plant glycans. In humans, *Oscillospira* was positively correlated with harder stools [51], and the abundant reduction in this genus was associated with inflammatory status [52]. If confirmed in swine, both of these properties could be useful during the weaning period to counteract some of the negative effects of solid-diet introduction, such as diarrhea and inflammation. Based on our analysis, *Oscillospira* was positively correlated with both ADG and BW. Other significant differences were detected in the tributyrin group for another genus, *Sutterella,* which could potentially affect gut metabolism. Indeed, Argüello et al. [53] have hypothesized the interfering with the gut colonization of pathogenic *Proteobacteria* by competition or by stimulation of the immune system. While the role of *Sutterella* is still unclear, the genus has been positively associated with average daily feed intake (ADF) [54]. Our results also confirm a positive correlation of this genus with ADG and ADF, though we did not detect significant differences in feed intake between the two animal groups.

Changes in fecal SCFA concentrations can reflect the different balance between groups of bacteria present in the gut microbiota. However, the SCFA concentration in stools must be considered as an approximation of gut microbiota metabolism products since, immediately after their production, enteric cells remove them from the intestinal lumen [55]. In spite of the changes detected in gut microbiota composition due to tributyrin, the determination of SCFA did not reveal significant differences between the two groups of animals, with the exception of a significant increase in isobutyrate. The higher amount of isobutyrate in the tributyrin group could indicate that the supplementation improved diet protein catabolism since this branched short-chain fatty acid is the result of valine degradation [56]. The increase in isobutyrate has been proposed as a parameter of diet protein utilization that characterizes the microbiota of high-feed efficiency pigs [23]. Little is known about the physiological impact of this branched short-chain fatty acid, even though isobutyrate has been shown to be involved in glucose and lipid metabolisms [57]. Interestingly, in our study *Oscillospira* and *Oscillibacter* showed a weak though significant positive correlation with fecal levels of isobutyrate.

Finally, in order to investigate changes in the functional capacity of gut microbiota as a result of dietary intervention, a functional metagenomics prediction approach was adopted. The analysis of the pathways of both animal groups using the KEGG reference pathways allowed us to highlight the reduction in carbohydrate metabolisms in the tributyrin group and, in particular, genes involved in sugar transport such as PTS transport systems. Conversely, in the same group of animals, genes involved in energy metabolism were enriched. The reduction in carbohydrate metabolism and, in particular, the sugar transport systems, seemed to be compensated by other metabolic pathways, such as those of amino acid catabolism, gluconeogenesis and the glyoxylate cycle. Commonly, bacteria use amino acid as an energy source after their deamination or decarboxylation. On the other hand, the increase in isobutyrate production could be ascribed to an increase in amino acid catabolic pathways.

Concerning the enrichment of genes involved in both gluconeogenesis and glyoxylate, it has been suggested that both pathways are used as a response to limited sources of fermentable carbohydrates. The glyoxylate shunt has been investigated in *E. coli*, *Salmonella* and other pathogens [58], though it is widespread among bacteria. The entire glyoxylate pathway of *Butyrivibrio proteoclasticus* is available in the KEGG database. Though we found no additional details, we can speculate that other species belonging to the genus *Butyrivibrio* can harbor genes encoding for this pathway as well as other intestinal bacteria. Accordingly, it is very difficult to hypothesize a putative implication of these pathways for the host’s health and performances. However, it is important to underline that these prediction analyses are based on genome sequencing data and, therefore, do not provide information concerning gene expression and its regulation. Conversely, tributyrin selected groups of bacteria that harbor genes coding for glyoxylate shunt enzymes. Further analysis is required, and a metabolomics approach could help in evaluating both the reduction in carbohydrate metabolisms and the increase in gluconeogenesis and glyoxylate pathways following tributyrin supplementation.

As far as we are aware, this is the first investigation focused on the effects of tributyrin supplementation on the gut microbiota of weaned piglets and demonstrating a relevant impact of this treatment on the composition and metabolic potential of fecal microbial communities. Thus, the beneficial effects of tributyrin may not be limited to supplementation of an extra source of energy to the enterocytes, which positively affects the integrity of the intestinal mucosa. Indeed, our results indicate that gut microbiota, in association with host-derived/related factors, may also play a role in exerting a positive impact on animal performances. The major limitation of this study is that the tested animals did not exhibit diarrhea episodes or health problems, yet our intent was to work in breeding conditions, without any challenge with pathogenic microorganisms. Nevertheless, the findings of the present study warrant further investigation to also elucidate the effects of tributyrin in artificial infection experiments mimicking swine enteric diseases.

## Figures and Tables

**Table 1:** Mean values of short-chain fatty acid (SCFA) concentrations of tributyrin (TRI) and control (CTR) piglet samples. Data are presented as mmol% ± SD.

	Acetate	Propionate	Isobutyrate	Butyrate	Isovalerate	Valerate
CTR (n = 12)	58.63 ± 6.15	23.93 ± 4.45	1.74 ± 0.91	9.93 ± 2.41	2.25 ± 1.37	3.51 ± 1.09
TRI (n = 12)	57.22 ± 3.35	22.72 ± 2.64	2.29 ± 0.82	10.84 ± 1.68	3.00 ± 1.24	3.93 ± 0.95
*p*-value	0.8852	0.2983	0.0269	0.4357	0.1410	0.5067

## Data Availability

Sequence files are available at the European Nucleotide Archive (ENA) database under the accession number PRJEB40653.

## Supplementary Materials

The following are available online at https://www.mdpi.com/2076-2607/9/3/584/s1: Table S1: Diet compositions of in vivo trial (% as fed basis) divided per control (CTR) and treatment group fed with 0.2% of tributyrin (TRI). Table S2: Differentially abundant phyla and families between control and tributyrin groups of piglets. Table S3: Differentially abundant genera between tributyrin and control groups of piglets. Table S4: Summary table of r correlation value and corresponding p value of Spearman correlation analysis.
