# Supplementary material for: Implications of Tributyrin on Gut Microbiota Shifts Related to Performances of Weaning Piglets
Source: Microorganisms. 2021 Mar 12;9(3):584. doi: 10.3390/microorganisms9030584 (PMC8001585; doi:10.3390/microorganisms9030584)
Supplement: Supplementary file 1 [file microorganisms-09-00584-s001.pdf]

Table S1. Diets composition of *in vivo* trial (% as fed basis) divided per control (CTR) and treatment group fed with 0.2% of tributyrin (TRI).

**Items**[1]	**CTR**	**TRI**
**Ingredients, % as fed basis**		
Barley, meal	25.15	25.15
Wheat, meal	19.41	19.36
Corn, flakes	14.03	13.83
Corn, meal	4.85	4.85
Soybean, meal	4.65	4.65
Soy protein concentrates	4.11	4.11
Biscuits, meal	4.00	4.00
Dextrose monohydrate	3.50	3.50
Wheat middlings	4.32	4.32
Whey protein concentrate	3.00	3.00
Fish, meal	2.50	2.50
Milk whey, powder	2.50	2.50
Coconut oil	1.00	1.00
Soybean oil	1.00	1.05
Plasma, meal	1.00	1.00
Organic Acids[2]	1.00	1.00
Dicalcium phosphate	0.85	0.85
Animal fats	0.70	0.70
L-Lysine	0.50	0.50
Benzoic acid	0.40	0.40
L-Threonine	0.35	0.35
DL-Methionine	0.35	0.35
Sodium Chloride	0.27	0.27
Vitamins[3]	0.25	0.25
L-Valine (96.5%)	0.15	0.15
L-Tryptophan	0.08	0.08
Flavouring[4]	0.04	0.04
Copper sulphate	0.04	0.04
Tributyrin[5]	-	**0.20**
**Calculated nutrient levels**[6]**, % as fed basis**		
Crude protein	16.92	16.90
Ether extract	5.06	5.16
Crude fibre	3.15	3.15
Ashes	5.10	5.10
DE[7] (Mcal/Kg)	3.43	3.43

[1]Ctrl: basal diet; Trib: basal diet supplemented with tributyrin (0.2%).

[2]Organic Acids: formic acid, sodium formate, sorbic acid, orthophosphoric acid, calcium formate, citric acid, and fumaric acid.

[3]Vitamins and vitamin-like compounds per kg: Vitamins and vitamin-like compounds per kg: Vitamin A, 10,000; Vitamin D3, 1000 IU; Vitamin E, 100 mg; Vitamin B1, 3 mg; Vitamin B2, 96.3 mg; Vitamin B6, 5.8 mg; Calcium D-pantothenate, 27 mg; Vitamin B12, 0.040 mg; Vitamin K3, 4.8 mg; Biotin, 0.19 mg; Niacinamide, 35 mg; Folic Acid, 1.4 mg. Choline Chloride 120 mg, Betaine Chloride 70 mg.

[5]Tributyrin (ACIFIS® Tri-B, New Feed Team srl, Italy).

[6]Nutrients content were calculated using Plurimix software (Fabermatica, CR, Italy).

[7]DE: digestible energy estimated following NRC (2012).

Table S2. Differentially abundant phyla and families between control and tributyrin groups of piglets.

Phylum	Log 2FC	Log CPM	P values	FDR
Actinobacteria	-41.335	15.129	0.0000000922	0.00000101
Cyanobacteria	21.171	13.499	0.00012201	0.00067107
Firmicutes	-10.634	19.775	0.00057935	0.0021243
Spirochaetae	24.989	10.801	0.006694	0.015076
Deferribacteres	16.739	82.193	0.0078265	0.015076
Proteobacteria	0.84114	15.851	0.0082234	0.015076
Chlamydiae	-1.495	88.122	0.071962	0.11308
Tenericutes	0.84124	12.176	0.21117	0.29036
Elusimicrobia	-0.61777	66.501	0.34364	0.42001
Bacteroidetes	0.012261	17.141	0.95887	0.98223
Fibrobacteres	-0.026921	10.277	0.98223	0.98223
Family				
Family XIII *Incertae Sedis*	-29.782	14.977	0.000000000004	0.000000000142
Coriobacteriaceae	-40.465	15.072	0.000000071213	0.000001246200
Lachnospiraceae	-19.987	17.765	0.167150000000	0.00015123
Erysipelotrichaceae	-2.084	15.722	0.172830000000	0.00015123
Not_assigned	23.597	13.6	0.217220000000	0.00015205
Peptococcaceae	-22.532	10.96	0.465780000000	0.00027171
VadinBB60	2.255	11.334	0.00015061	0.00066084
Human gut metagenome	35.149	95.942	0.00015225	0.00066084
Peptostreptococcaceae	-25.271	11.209	0.00016993	0.00066084
Campylobacteraceae	16.726	14.157	0.00077039	0.0026964
Spirochaetaceae	29.209	10.947	0.0018182	0.0057853
Deferribacteraceae	20.601	8.355	0.0027565	0.0080397
Mycoplasmataceae	16.771	70.548	0.00993	0.026735
Neisseriaceae	-26.369	94.771	0.014295	0.035738

Table S3. Differentially abundant genera between tributyrin and control groups of piglets.

Genus	log2 FC	log CPM	P values	FDR
Mogibacterium	-38.395	14.043	0.00000000013	0.00000000808
Collinsella	-2.424	13.055	0.00166310000	0.05155600000
Butyrivibrio	41.845	95.802	0.00422660000	0.00873500000
Atopobium	-38.259	11.221	0.00058480000	0.09064500000
Denitrobacterium	-35.554	98.305	0.06521200000	0.78701000000
Not assigned	24.174	14.269	0.07616200000	0.78701000000
Oscillibacter	24.011	13.597	0.27518000000	0.00024373
Syntrophococcus	-27.614	16.332	0.32008000000	0.00024806
Peptococcus	-21.588	10.729	0.45169000000	0.00031116
Mucispirillum	26.295	85.381	0.75976000000	0.00047105
Roseburia	-30.369	14.359	0.90751000000	0.0005115
Desulfovibrio	14.469	14.825	0.00010357	0.00053511
Marvinbryantia	-23.647	9.101	0.00013585	0.00064792
Campylobacter	19.874	14.277	0.00015231	0.0006745
Treponema	3.592	11.598	0.00030256	0.0012506
Blautia	-26.132	13.826	0.00044296	0.0016767
Enterorhabdus	-18.561	70.193	0.00045974	0.0016767
Oscillospira	17.249	10.682	0.00056126	0.0019332
Mycoplasma	19.736	7.114	0.00061427	0.0020045
Subdoligranulum	22.653	82.743	0.00090021	0.0027513
Sutterella	16.524	11.934	0.00093191	0.0027513
Oribacterium	-15.643	84.969	0.0037963	0.010699
Mitsuokella	-17.117	11.875	0.0044539	0.012006
Uncultured bacterium	12.384	14.428	0.0059718	0.015427
Oxalobacter	16.206	74.623	0.0071181	0.017653
Selenomonas	-18.234	13.39	0.007903	0.018846
Uncultured *Clostridiales* bacterium	12.572	71.685	0.009513	0.021845
Pseudoflavonifractor	12.846	10.587	0.010833	0.023988
Megasphaera	-13.065	14.193	0.015534	0.033211
Pseudobutyrivibrio	1.746	13.097	0.017661	0.0365

Table S4. Summary table of r correlation value and corresponding p value of Spearman correlation analysis between performance and physiological parameters and significantly different genera between the two dietetic groups. Light blue colour indicates negative correlations that are significant while green colour underlines the significant positive correlations. ADG (Average Daily Gain); BW (Body Weight); TC (Total Cholesterol); LDL (Low-Density Lipoproteins). The correlation coefficient r and the p value are reported for each comparison, separated by a semicolon.

Genus	ADG g/d r; p values	BW Kg r; p values	Isobutyrate mmol/100g r; p values	Urea mmol/L r; p values	Glucose mmol/L r; p values	TC mmol/L r; p values	LDL mmol/L r; p values
Atopobium	-0.56; 0.004695	-0.36; 0.080758	-0.45; 0.026202	0.48; 0.017620	-0.38; 0.070475	-0.48; 0.018906	-0.32; 0.129986
Blautia	-0.33; 0.110485	-0.08; 0.710075	-0.26; 0.211836	0.53; 0.007434	-0.26; 0.226473	-0.35; 0.096325	-0.40; 0.052052
Butyrivibrio	0.45; 0.027116	-0.13; 0.544927	0.12; 0.574638	-0.43; 0.036693	0.33; 0.119596	0.65; 0.000547	0.62; 0.001334
Collinsella	-0.81; 0.000002	-0.43; 0.037622	-0.37; 0.071159	0.42; 0.043261	-0.10; 0.657724	-0.41; 0.046558	-0.38; 0.064216
Desulfovibrio	0.59; 0.002207	0.27; 0.198862	0.32; 0.124164	-0.44; 0.032445	0.41; 0.046095	0.40; 0.049700	0.31; 0.134476
Enterorhabdus	-0.44; 0.030425	-0.07; 0.754478	-0.21; 0.334177	0.34; 0.099988	0.04; 0.862055	-0.11; 0.623147	-0.05; 0.807862
Marvinbryantia	-0.53; 0.007230	0.09; 0.674910	0.03; 0.884522	0.60; 0.001988	-0.18; 0.408096	-0.53; 0.008369	-0.45; 0.028258
Mitsuokella	-0.49; 0.014680	-0.43; 0.036344	-0.71; 0.000089	0.09; 0.676468	-0.02; 0.917999	-0.19; 0.363813	-0.07; 0.729783
Mogibacterium	-0.68; 0.000258	-0.40; 0.051553	-0.25; 0.240308	0.62; 0.001262	-0.28; 0.183451	-0.59; 0.002626	-0.45; 0.027577
Mucispirillum	0.58; 0.003242	0.10; 0.628500	0.20; 0.335523	0.39; 0.055987	-0.02; 0.932360	0.44; 0.031744	0.38; 0,070850
Oscillospira	0.61; 0.001660	0.55; 0.005515	0.44; 0.029383	-0.45; 0.024860	0.22; 0.313874	0.37; 0.073899	0.24; 0.266083
Oscillibacter	0.53; 0.007303	0.44; 0.030463	0.57; 0.003607	-0.27; 0.207601	0.10; 0.635814	0.30; 0.156156	0.25; 0.237668
Peptococcus	-0.65; 0.000619	-0.04; 0.863847	-0.10; 0.633287	0.67; 0.000305	-0.23; 0.290425	-0.56; 0.004495	-0.45; 0.028991
Sutterella	0.54; 0.006677	0.33; 0.110622	0.39; 0.056479	-0.40; 0.051494	0.34; 0.100997	0.4; 0.049245	0.26; 0.225972
Syntrophococcus	-0.47; 0.019027	-0.24; 0.259429	-0.42; 0.042901	0.38; 0.066235	-0.26; 0.212204	-0.28; 0.182888	-0.10; 0.630245
